# Between algorithmic alerts and bodily sensations: a qualitative study of post-PCI patients' experiences with wrist-worn heart health information

**DOI:** 10.3389/fcvm.2026.1926958

**Published:** 2026-10-19

**Authors:** Angang Tong, Ruiqi Luo, Pan Jin, Cuihuan Wu

**Affiliations:** Department of Cardiology, Union Hospital, Tongji Medical College, Huazhong University of Science and Technology, Wuhan, China

**Keywords:** heart disease, patient-generated health data, percutaneous coronary intervention, qualitative research, self-management, smart wrist-worn devices, wearable technology

## Abstract

**Background:**

Smart wrist-worn devices are increasingly used by patients after percutaneous coronary intervention (PCI) to monitor heart rate, rhythm-related alerts, sleep, activity, and other health-related data. However, limited qualitative evidence exists on how post-PCI patients interpret and respond to such information in daily life.

**Objective:**

This study aimed to explore how patients with coronary heart disease after PCI understand, evaluate, and respond to heart rate, heart rhythm, and health-related information generated by smart wrist-worn devices.

**Methods:**

A qualitative descriptive design was adopted. From January to May 2026, purposive sampling was used to recruit 23 post-PCI patients who had used smartwatches or smart bands in daily life. Semi-structured interviews were conducted, audio-recorded, transcribed verbatim, and analyzed using thematic analysis. Data collection and analysis were conducted concurrently until data saturation was achieved.

**Results:**

Four themes were developed: (1) cardiac status became continuously datafied; (2) calibrating risk between alerts and sensations; (3) device information awaited medical interpretation; and (4) from self-management to disease burden. Patients did not simply accept or reject device-generated data. Instead, they interpreted alerts by comparing them with bodily sensations, previous cardiac events, stent implantation experiences, follow-up results, and healthcare professionals' explanations. Device information could enhance awareness of medication use, follow-up, and lifestyle management. However, when alerts lacked medical interpretation or follow-up response, they also increased uncertainty, worry, repeated self-monitoring, and perceived disease burden.

**Conclusions:**

Smart wrist-worn devices may support daily observation and clinician–patient communication in post-PCI management, but they should not be regarded as substitutes for clinical examinations or professional judgment. Clearer interpretive boundaries, stratified guidance, and follow-up response pathways are needed to help patients appropriately understand and act on device-generated information.

## 1 Introduction

After patients with coronary heart disease (CHD) undergo percutaneous coronary intervention (PCI), localized coronary artery stenosis or acute ischemic conditions may be improved; however, the progression of atherosclerosis and the risk of major adverse cardiovascular events do not cease (1). The focus of post-PCI management therefore shifts from acute-phase revascularization to long-term secondary prevention, involving adherence to antiplatelet and lipid-lowering therapy, risk factor control, symptom recognition, and lifestyle modification (2). Unlike healthcare professionals, who primarily assess risk based on examination findings and clinical guidelines, most patients understand their postoperative condition through everyday bodily experiences. Chest tightness, palpitations, or changes in sleep may be regarded as ordinary discomfort, but they may also be interpreted as signs of recurrent ischemia or stent-related problems. Thus, post-PCI management is not only a matter of follow-up, medication use, and risk factor control, but also concerns how patients continuously perceive bodily changes in daily life, interpret the risk-related significance of these changes, and accordingly decide whether to seek medical help, adjust their behaviors, or continue observation (3). In other words, postoperative risk management does not occur solely within medical settings; it also unfolds through patients' ongoing interpretation of their bodily experiences and their subsequent judgments about action.

The use of smart wrist-worn devices is changing the ways in which post-PCI patients access information related to cardiac health (4). Devices such as smartwatches and smart bands can continuously collect data on heart rate, rhythm-related signals, physical activity, sleep, blood oxygen, and exercise recovery through technologies such as photoplethysmography, single-lead electrocardiography, accelerometers, blood oxygen monitoring, and sleep monitoring. Built-in algorithms then generate abnormality alerts, trend changes, health scores, or behavioral recommendations (5). Unlike examination results that are periodically generated by healthcare institutions during conventional follow-up and interpreted by healthcare professionals, information from wrist-worn devices is continuous, immediate, and directly accessible to patients. As a result, heart rate, heart rhythm, and health-related data extend beyond the consultation room and examination reports into patients' everyday lives (6). Patients are therefore not only informed of their physical recovery during follow-up visits, but also repeatedly encounter bodily data and algorithmic alerts presented by the device while walking, resting, sleeping, exercising, or experiencing mild discomfort.

However, heart rate, heart rhythm, and health-related data generated by wrist-worn devices are not equivalent to clinical diagnoses or treatment recommendations (7). Elevated heart rate, rhythm abnormality alerts, changes in sleep, or reduced activity tolerance may reflect normal physiological fluctuations, but they may also be interpreted by patients as postoperative risk signals (8). For post-PCI patients, such information is often not viewed as neutral health data; rather, it is reinterpreted in relation to their experience of stent implantation, previous episodes of chest pain or myocardial infarction, follow-up results, and physicians' recommendations. When device alerts are consistent with bodily sensations, patients may regard them as confirmation of their symptoms (9): When the device indicates an abnormality but the patient has no obvious discomfort, patients may be uncertain about whether action is needed. Conversely, when bodily discomfort occurs without a corresponding device alert, patients may question the reliability of the device or reassess their own sensations (10). Therefore, information generated by smart wrist-worn devices places patients within an interpretive process shaped jointly by algorithmic alerts, bodily sensations, and prior medical experiences.

Existing studies on smart wrist-worn devices in cardiovascular care have mainly focused on heart rate measurement, rhythm abnormality detection, remote monitoring, and the integration of patient-generated health data (11, 12). These studies have established important technical and clinical foundations, but have largely emphasized device accuracy, algorithm performance, data integration, and healthcare professionals' use of wearable-generated information. Qualitative and mixed-methods studies have also examined patients' experiences of wearable and mobile health technologies in atrial fibrillation, heart failure, and cardiac rehabilitation, identifying issues related to trust, anxiety, self-management, and communication with healthcare professionals (13–15). However, the post-PCI context may involve a distinct interpretive process. Patients have undergone a tangible revascularization procedure and may experience symptomatic relief, yet they remain exposed to long-term atherosclerotic progression and recurrent cardiovascular risk. Consequently, device-generated alerts may be interpreted in relation to stent patency, recurrent coronary occlusion, or another cardiac event, even though consumer wearables cannot directly assess these conditions. How the experience of PCI and stent implantation shapes patients' interpretation of and response to wearable-generated information remains insufficiently understood (16).

Understanding this process from the patient perspective is important for developing patient-centered digital cardiovascular management. If algorithmic alerts can be understood by patients and connected with appropriate clinical interpretation and follow-up support, smart wrist-worn devices may help strengthen symptom vigilance, awareness of follow-up, and consciousness of lifestyle management (17). Conversely, if device information is unclear, inconsistent with patients' bodily sensations, or lacks interpretation and response from healthcare professionals, it may increase patients' uncertainty, excessive worry, repeated self-monitoring, and management burden. Therefore, evaluating the value of smart wrist-worn devices in post-PCI management should not focus only on data accuracy and technological capability, but should also consider how patients interpret and respond to such information in their daily lives.

Based on this rationale, the present study adopted a qualitative research design to explore how patients with CHD after PCI understand and respond to heart rate, heart rhythm, and health-related data generated by smart wrist-worn devices. This study focuses on how patients compare algorithmic alerts with bodily sensations, previous cardiac events, experiences of stent implantation, follow-up results, and healthcare professionals' recommendations, as well as how these understandings influence their expectations for medical care, awareness of medication use and follow-up, symptom observation, and postoperative self-management behaviors. The findings may provide patient-side evidence to inform information presentation, clinician–patient communication, and digital follow-up support in the management of post-PCI patients using smart wrist-worn devices.

## 2 Methods

### 2.1 Study design

This study adopted a qualitative descriptive research design (18), to explore how patients with coronary heart disease (CHD) after percutaneous coronary intervention (PCI) understood, responded to, and experienced self-management in relation to heart rate, heart rhythm, and health-related data generated by smart wrist-worn devices during device use. A qualitative descriptive design is appropriate for presenting patients' everyday experiences, subjective interpretations, and behavioral responses in a manner that remains close to the data itself. It helps reveal how patients interpret the risk-related meaning of their postoperative condition in relation to device alerts, bodily sensations, previous disease experiences, follow-up results, and explanations provided by healthcare professionals.

This study did not evaluate the diagnostic value of smart wrist-worn devices for post-PCI clinical outcomes, stent patency, or coronary restenosis. Rather, it focused on how patients understood and used information generated by consumer-grade smart wrist-worn devices, and how such information influenced their risk judgments, expectations for medical care, and self-management behaviors.

### 2.2 Participants and setting

Purposive sampling was used to recruit participants from January 2026 to May 2026 in the Department of Cardiology of Union Hospital, Tongji Medical College, Huazhong University of Science and Technology, Wuhan. Participants were recruited from cardiology outpatient clinics, cardiac rehabilitation clinics, and post-PCI follow-up populations.

The inclusion criteria were as follows: (1) clinically diagnosed with CHD and had undergone PCI; (2) in the home recovery or long-term follow-up stage after PCI; (3) had previously used or were currently using smart wrist-worn devices, such as smartwatches or smart bands, and viewed heart rate, heart rhythm alerts, sleep, physical activity, blood oxygen, or electrocardiography-related information in daily life; (4) were able to clearly express their experiences and views; and (5) voluntarily agreed to participate in the study and provided written informed consent.

The exclusion criteria were as follows: (1) severe cognitive impairment, mental disorder, or communication impairment that prevented completion of the interview; (2) being in an acute cardiovascular event, severe heart failure, or another clinical condition unsuitable for interview participation; and (3) wearing a smart wrist-worn device but never viewing or using the relevant health data, making it impossible to provide experience related to the research questions.

To avoid conflating information generated by different types of devices, the smart wrist-worn devices used by participants and the main indicators they attended to were recorded separately during data collection and analysis. These included: (1) users of smartwatches with electrocardiography or heart rhythm abnormality alert functions; (2) patients who mainly attended to heart rate and post-exercise heart rate changes; (3) patients who mainly attended to health-related indicators such as sleep, physical activity, and blood oxygen; and (4) patients who had received device-generated abnormality alerts or had sought medical consultation because of device alerts. This information was used to facilitate understanding of how different patients interpreted device alerts and how their responses varied.

During sampling, the principle of maximum variation was considered. Patients with diverse characteristics were included as far as possible in terms of sex, age, educational level, time since PCI, disease severity, type of smart wrist-worn device, duration of device use, and frequency of use, in order to obtain rich and diverse data. A total of 23 patients with CHD after PCI were interviewed in this study. Recruitment continued until the main prespecified dimensions of variation had been adequately represented and additional interviews no longer generated substantively new codes or themes.

Maximum variation sampling was used to broaden the range of patient experiences represented in the data rather than to examine causal relationships between participant characteristics and study outcomes. Demographic, clinical, and device-related differences were treated as contextual features of participants' experiences. During thematic analysis, the research team examined recurrent patterns across participants while also retaining divergent experiences that contributed to a more comprehensive understanding of the phenomenon.

### 2.3 Research team

Members of the research team had backgrounds in cardiovascular nursing, chronic disease management, and qualitative research methods. The principal investigator was responsible for study design, participant recruitment, interview implementation, and data analysis. Researchers involved in data analysis had received training in qualitative research methods and were familiar with semi-structured interviews, interview transcript preparation, coding, and thematic analysis.

The interviewer had a professional background in nursing and was familiar with follow-up care and health education for post-PCI patients. Before the interviews, the interviewer clearly explained their identity as a researcher to the patients and emphasized that the interview content would be used only for scientific research and would not affect patients' subsequent medical treatment or nursing services. If the interviewer had prior contact with a patient through clinical care or follow-up, the research team attempted to minimize the influence of the researcher's identity and professional position on patients' expressions by maintaining open-ended questioning, avoiding leading interpretations, and recording reflexive memos during the interview and analysis processes.

Throughout the study, the team held regular discussions to repeatedly review the interview guide, initial coding, category development, theme naming, and data interpretation. When researchers had different understandings during coding and interpretation, the team checked these interpretations by returning to the original interview transcripts, referring to field notes, and conducting group discussions. These procedures were used to reduce bias arising from the interpretation of a single researcher and to enhance the stability and credibility of the analytic findings.

### 2.4 Data collection

Data were collected through semi-structured in-depth interviews. The interview guide was developed through a structured process. First, the research team identified the main interview domains based on the study aim and literature concerning post-PCI secondary prevention, patient-generated health data, smart wrist-worn device use, and patient self-management. These domains included the circumstances of device adoption, information routinely attended to, interpretation and management of abnormal alerts, concordance or discordance between device data and bodily sensations, the influence of previous illness and treatment experiences, changes in postoperative risk perception, effects on self-management, perceived benefits and burdens, and expectations for healthcare support. Based on these domains, the research team drafted an initial set of open-ended questions arranged from general device-use experiences to specific alert-related situations. The draft interview guide was subsequently reviewed by a multidisciplinary group of experts with expertise in cardiovascular nursing, clinical nursing, and qualitative research. The experts assessed the relevance, clarity, neutrality, sequencing, and comprehensibility of the questions. Based on their feedback, overlapping questions were integrated, potentially leading wording was revised, and the sequence of questions was adjusted. The final interview guide comprised nine open-ended questions, with follow-up questions asked flexibly according to participants' responses. Further details regarding the development and rationale of the interview guide are provided in Supplementary File 1.

Before each formal interview, the researcher explained the study purpose, interview content, audio-recording procedure, confidentiality measures, and principle of voluntary participation. Interviews were conducted after written informed consent had been obtained. At the same time, the researcher used a general information form to collect participants' demographic and disease-related information, including age, sex, educational level, marital status, occupational status, time since PCI, reason for PCI or clinical background, comorbidities, medication use, participation in cardiac rehabilitation, previous readmission or abnormal follow-up findings, type of smart wrist-worn device, duration of use, frequency of use, and the main health dsata attended to. When necessary, and with patients' consent, the researcher reviewed selected clinical records to verify disease diagnosis, PCI treatment background, and major comorbidities (see Supplementary File 1 for the interview guide).

All interviews were conducted by researchers trained in qualitative interviewing. Interviews were conducted in quiet, private, and convenient settings for patients, such as an independent consultation room in the outpatient clinic, a teaching room in the ward, or a private online environment. Before each interview, the researcher established basic rapport with the patient and encouraged them to describe their real experiences in relation to specific life situations. During the interview, the researcher asked appropriate follow-up questions based on the patient's responses to obtain richer narratives. For example, when patients mentioned “high heart rate,” “irregular heart rhythm,” “poor sleep,” “low blood oxygen,” “device alarm,” “worrying that the stent was blocked,” or “feeling more nervous after looking at the data,” the researcher further asked about their bodily sensations at the time, risk judgments, coping strategies, and whether they had sought help from healthcare professionals.

Each interview lasted 30–60 min, with an average duration of 48 min. With patients' consent, all interviews were audio-recorded and transcribed verbatim as soon as possible after completion. The researcher recorded field notes during and after the interviews, including patients' tone of voice, pauses, emotional responses, nonverbal expressions, and the interview context. Data collection and data analysis were conducted concurrently. By the 20th interview, no substantive new codes or themes related to the use of smart wrist-worn devices, understanding of postoperative risk, or self-management experiences had emerged (19). The research team then conducted three additional interviews to confirm data saturation. Recruitment was stopped after the team determined that data saturation had been achieved.

### 2.5 Data analysis

Thematic analysis was used to analyze the interview data, and NVivo software was used for data management. The analysis process followed the steps of thematic analysis proposed by Braun and Clarke (20). First, the researchers repeatedly read the interview transcripts and field notes to become familiar with patients' overall narratives regarding smart wrist-worn device use, bodily sensations, data interpretation, and postoperative management. Second, the interview transcripts were coded line by line using open coding. Meaning units relevant to the research questions were extracted, and initial codes were developed in a manner that remained as close as possible to patients' own words. Third, the researchers compared, merged, and categorized the initial codes to form categories with similar meanings. Subsequently, the research team repeatedly discussed the relationships among categories and further refined potential themes and subthemes. Finally, the research team returned to the original interview data to examine whether the themes adequately explained patients' experiences, and revised the theme names and meanings accordingly.

During the analysis, particular attention was paid to how patients compared data provided by smart wrist-worn devices with their own bodily sensations, previous disease experiences, PCI treatment experiences, follow-up results, and healthcare professionals' recommendations. For the same type of device alert, different patients might show different responses, such as vigilance, neglect, repeated confirmation, seeking medical explanation, or temporary suspension of action. The research team compared these differences during analysis and further interpreted the underlying logic of risk understanding and self-management.

To enhance analytic consistency, at least two researchers independently read and coded selected interview transcripts, and then compared and discussed the coding results. For sections in which coding was inconsistent or interpretations of themes differed, the research team reached consensus by returning to the original data, reviewing field notes, and conducting group discussions. Analytic memos were written throughout the analysis to record changes in researchers' understanding of code meanings, theme boundaries, and differences in patient experiences. The final themes were used to present the experiential structure through which patients with CHD after PCI understood their cardiac status, postoperative risk, and self-management in the context of smart wrist-worn device use.

### 2.6 Rigor

The quality of this study was ensured in terms of credibility, dependability, confirmability, and transferability. In relation to credibility, researchers used semi-structured in-depth interviews to collect data and encouraged patients to fully describe specific experiences through open-ended questions and follow-up probing. All interview recordings were transcribed verbatim, and the research team analyzed both interview transcripts and field notes to better understand the contexts of patients' narratives. During data analysis, multiple researchers participated in coding and theme discussions, and discrepant codes and interpretations were repeatedly checked until consensus was reached.

In relation to dependability, the research team maintained relatively consistent interview procedures and analytic processes throughout data collection and analysis. The interview guide was discussed and revised before formal use and was moderately refined during data collection based on interview content. In relation to confirmability, the research team retained interview recordings, transcripts, field notes, coding frameworks, analytic memos, and records of team discussions, thereby forming a complete audit trail that allowed the findings to be traced back to the original data. In relation to transferability, this study provided detailed descriptions of participant sources, inclusion and exclusion criteria, sample characteristics, data collection procedures, and data analysis methods, enabling readers to judge the applicability of the findings to other post-PCI patient populations or smart health device use contexts.

In addition, this study was reported with reference to the Consolidated Criteria for Reporting Qualitative Research (COREQ) (21). Particular attention was paid to presenting the characteristics of the research team, participant recruitment, data collection context, interview process, data analysis steps, theme development process, and original interview quotations, in order to improve the completeness and transparency of the research process and reporting of findings (Supplementary File 2).

### 2.7 Ethical considerations

This study was approved by the Ethics Committee of Union Hospital, Tongji Medical College, Huazhong University of Science and Technology (Approval No. 2025XHYN093). Before the interviews, the researcher explained the study purpose, study procedures, audio-recording arrangements, data storage methods, confidentiality measures, and the voluntary nature of participation. All participants provided written informed consent before taking part in the study. Participants were informed that they had the right to withdraw from the study at any stage, and that withdrawal would not affect their subsequent medical treatment or nursing services.

To protect participants' privacy, all research data were anonymized using codes such as P1, P2, and P3. Identifiable personal information, including names, contact details, and addresses, was removed from interview transcripts and research reports. All data were used only for research analysis and academic reporting, and were securely stored by the research team.

## 3 Results

### 3.1 Characteristics of participants

A total of 23 patients with coronary heart disease (CHD) after percutaneous coronary intervention (PCI) were included in this study, including 15 men and 8 women, aged 52–74 years (Supplementary File 3). The time since PCI ranged from 4 months to 5 years. The clinical backgrounds for PCI included acute myocardial infarction, acute coronary syndrome, unstable angina, and elective PCI for stable CHD. Most patients had comorbid hypertension, diabetes mellitus, or hyperlipidemia. All patients had previously used or were currently using smart wrist-worn devices, including smartwatches and smart bands. The duration of device use ranged from 4 months to 3 years. Patients mainly attended to data such as heart rate, heart rhythm or electrocardiography-related information, sleep, step count, physical activity, and blood oxygen. Among them, 18 patients had received device-generated alerts for elevated heart rate, rhythm abnormalities, abnormal sleep, or abnormal blood oxygen. The sample showed variation in age, sex, time since PCI, disease background, device type, duration of use, and indicators of concern, thereby providing relatively rich data for understanding how post-PCI patients interpreted and responded to information from smart wrist-worn devices (Table 1).

**Table 1:** Revised summary.

Characteristic	Summary
Age, years, mean ± SD	62.4 ± 6.5
Age range, years	52–74
Sex, male/female	15 (65.2%)/8 (34.8%)
Educational level	
Primary school	5 (21.7%)
Junior high school	7 (30.4%)
High school/technical secondary	4 (17.4%)
Junior college	3 (13.0%)
Bachelor's degree	4 (17.4%)
Time since PCI, months	
Mean ± SD	21.0 ± 16.7
Range	4–60
PCI-related clinical background	
Acute myocardial infarction	6 (26.1%)
Acute coronary syndrome	6 (26.1%)
Unstable angina	5 (21.7%)
Elective PCI for stable CHD	6 (26.1%)
Major comorbidities^a^	
Hypertension	15 (65.2%)
Diabetes mellitus	7 (30.4%)
Hyperlipidemia	8 (34.8%)
Use of *β*-blockers	21 (91.3%)
Device type	
Huawei smartwatch	8 (34.8%)
Huawei smart band	4 (17.4%)
Xiaomi smart band	6 (26.1%)
Apple Watch	5 (21.7%)
Duration of device use, months	
Mean ± SD	16.0 ± 10.5
Range	4–36
Previous device-generated abnormality alert	18 (78.3%)
Attended to ECG-related information	5 (21.7%)

a Participants could report more than one comorbidity; therefore, percentages do not sum to 100%.

### 3.2 Themes

Four themes were developed in this study: (1) cardiac status became continuously datafied; (2) calibrating risk between alerts and sensations; (3) device information awaited medical interpretation; and (4) from self-management to disease burden. Overall, smart wrist-worn devices did not simply provide monitoring results. Rather, they made heart-related information continuously visible in patients' daily lives and prompted patients to compare device data with previous disease experiences, bodily sensations, follow-up results, and healthcare professionals' recommendations. The thematic structure of these four themes is presented in Figure 1.

**Figure 1:**
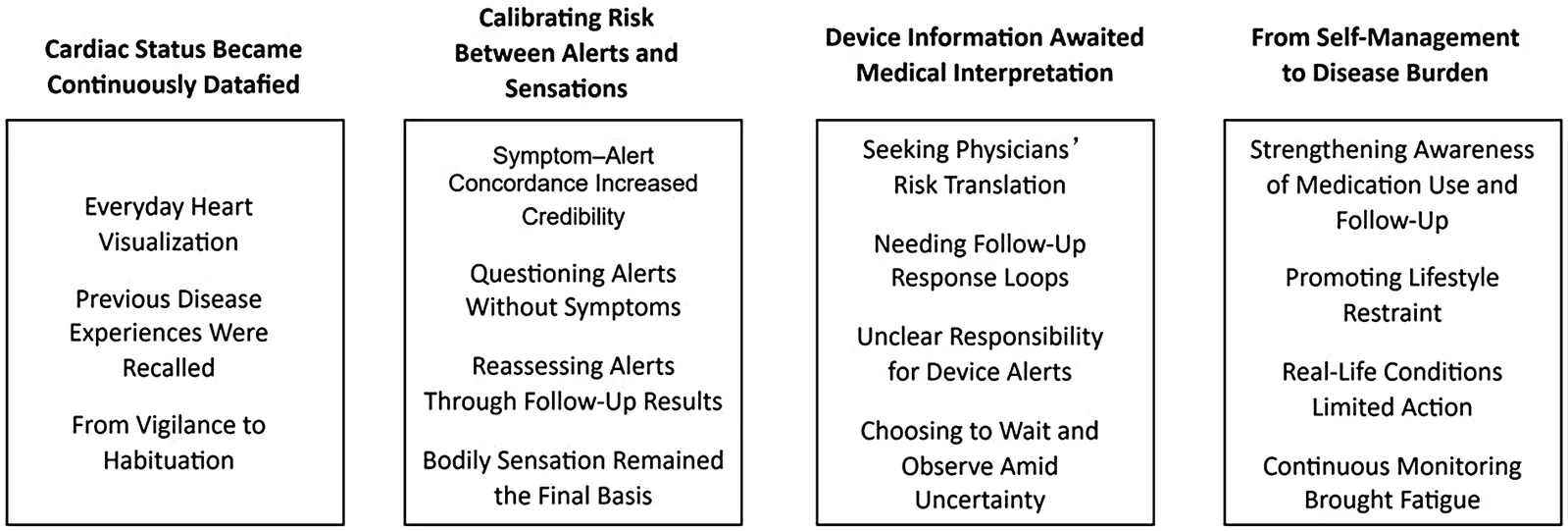
Thematic structure of the study results.

#### 3.2.1 Cardiac status became continuously datafied

##### 3.2.1.1 Everyday heart visualization

Smart wrist-worn devices enabled patients to encounter heart rate, heart rhythm, and health-related information more frequently in daily life. In the past, patients usually learned about their postoperative recovery through follow-up examinations or physicians' explanations. Now, heart rate, sleep, and activity information repeatedly appeared on their watch or mobile phone interface, making patients feel that their cardiac status was being continuously brought to their attention.

P8: “Before, I would only think about it when I felt uncomfortable somewhere. Now the watch keeps showing heart rate, sleep, and things like that. It shows them in the morning and at night. After seeing them so often, I feel as if it is constantly reminding me.”

Device alerts also changed the way patients observed their bodies. Mild increases in heartbeat, fatigue, or changes in sleep might not have previously received much attention. However, when these sensations were linked with device alerts, patients would reassess whether they were related to cardiac recovery.

P3: “Sometimes I didn't feel much myself. … Once it suddenly reminded me that my heart rate was high, so I stopped to check whether I had just been tired, or whether my heart was becoming a bit unstable again.”

Wrist-worn devices also extended cardiovascular monitoring from the hospital into everyday life. Patients might see relevant information while walking, sleeping, or after exercising. As a result, they were no longer merely waiting for follow-up results, but were continuously encountering and interpreting their cardiac status during daily activities.

P17: “Now I don't have to wait until I go to the hospital to know. Sometimes I just check it casually, and then I have some idea in my mind.”

##### 3.2.1.2 Previous disease experiences were recalled

Device-generated abnormality alerts easily evoked patients' previous disease experiences. For patients who had experienced chest pain, emergency admission, or PCI treatment, these alerts were not merely technical information; rather, they reminded them of the pain, fear, and medical care process they had previously experienced.

P2: “Once it reminds me that something is abnormal, I get scared and wonder whether something is going to happen.”

The experience of stent implantation also influenced how patients interpreted alerts. Some patients linked changes in heart rate or rhythm with whether the stent was stable or whether the blood vessel was blocked again. Although the device itself could not assess stent status, patients still interpreted this information within the post-PCI context.

P15: “After all, I had the procedure and a stent was put inside. Once it says something is abnormal, I wonder whether there is something wrong again with that blood vessel.”

Previous disease experiences also increased patients' sensitivity to alerts. Some patients had previously experienced situations in which symptoms were “not serious at first” but later turned out to indicate a major problem. Therefore, when facing device alerts after PCI, they became more cautious and worried about once again ignoring early abnormalities.

P12: “Last time, at the beginning I didn't take it too seriously. Later, after the examination, I found out it was not a small issue. … So I learned to pay more attention.”

##### 3.2.1.3 From vigilance to habituation

When patients first saw device-generated abnormality alerts, they often became noticeably anxious. In particular, when heart rate abnormalities, rhythm alerts, or sleep abnormalities appeared for the first time, patients tended to interpret them as new cardiac problems and quickly became more vigilant.

P6: “When I first saw that alert, I really felt quite nervous.”

As the duration of device use increased, if the device occasionally issued alerts but the patient had no chest pain, chest tightness, palpitations, or activity limitation, patients gradually became less anxious. At this point, bodily sensations became the main basis for judging the importance of the alert.

P6: “Later I found that it sometimes gave alerts anyway. After it happened more often, I was not as nervous as I was at the beginning.”

Whether healthcare professionals emphasized device information also affected the extent to which patients took it seriously. If physicians did not explain or mention it, patients might gradually regard device alerts as ordinary health reminders rather than medical problems requiring serious attention.

P21: “The doctor never specifically said there was any problem with the watch data, so I didn't take it too seriously.”

#### 3.2.2 Calibrating risk between alerts and sensations

##### 3.2.2.1 Symptom–alert concordance increased credibility

When device alerts coincided with bodily symptoms such as chest tightness, palpitations, or fatigue, patients were more likely to consider the alerts credible and interpret them as corroborating their perceived bodily changes.

P18: “Recently, when I walk a bit faster, I do feel a little tightness in my chest, and the watch also reminds me that my heart rate is high. So I feel that maybe it is not giving alerts randomly. It is indeed somewhat useful.”

Activity tolerance was also an important basis for patients to interpret alerts. Whether climbing stairs was strenuous, whether they became tired easily while walking, and whether heart rate increased markedly after exercise were all used by patients to judge whether device alerts were related to their postoperative condition.

P7: “I didn't feel it before either. Later the watch reminded me, and I felt that I still needed to pay attention and could not run like before.”

Device alerts also made vague discomfort more specific. Patients might initially only feel somewhat panicked or uncomfortable and be unable to identify what was wrong. However, after seeing an elevated heart rate, they were more inclined to believe that the sensation might be related to the heart.

P1: “At first I just felt a little palpitation, and I couldn't really say what was wrong. Then I saw that the heart rate on the watch was also high. … I didn't dare to completely ignore it.”

##### 3.2.2.2 Questioning alerts without symptoms

When the device showed an abnormality but patients had no obvious discomfort, they often found it difficult to understand the meaning of the alert. For them, chest pain, palpitations, or shortness of breath remained important bases for judging cardiac problems. If the body felt normal, abnormal alerts instead caused confusion.

P3: “That day it reminded me that something was abnormal, but I felt perfectly fine. Was it that I didn't feel it, or was the device measuring inaccurately?”

This uncertainty did not mean that patients completely rejected the device. Rather, they were unable to determine whether the alert represented a real risk, a measurement error, or a normal physiological fluctuation. In the absence of explanation, device alerts could instead increase uncertainty.

P5: “Sometimes when it gives an alert, I wonder whether I didn't wear the watch properly and it is reporting randomly.”

In the absence of symptoms, some patients did not seek medical care immediately. Instead, they chose to continue observing or to ask the doctor during the next follow-up visit. This indicates that device-generated abnormality alerts did not necessarily trigger action; whether patients felt bodily abnormalities also mattered.

P20: “If it is just one alert, I may watch it first. If it keeps happening later, or if my chest also feels uncomfortable, then I will ask the doctor.”

##### 3.2.2.3 Reassessing alerts through follow-up results

Patients actively compared device alerts with formal follow-up results. Electrocardiography, blood pressure and lipid levels, echocardiography, coronary-related examinations, and physicians' explanations remained the main bases for patients to judge their cardiovascular status, and were often used to reassess device alerts.

P10: “At the last follow-up, the doctor said things were still okay, so when the watch showed an abnormal alert, I started wondering. Which one is accurate, the watch or the examination? I really can't judge it myself.”

When device alerts were consistent with previous physicians' reminders or follow-up problems, patients were more likely to accept them. For example, if a physician had previously reminded a patient to control blood lipids or increase activity, and the device also indicated insufficient activity, the patient would connect the two and interpret them together.

P11: “The doctor had said before that I needed to control my blood lipids, and the watch also reminded me that I was not active enough.”

When device alerts were inconsistent with examination results, patients easily experienced a sense of contradiction. Most patients still trusted physicians and examination reports more, but device alerts disturbed their previously stable judgments and made them question again whether a problem existed.

P17: “The examination report says there is no major problem, but the watch says something is abnormal. Which one should I listen to?”

##### 3.2.2.4 Bodily sensation remained the final basis

Although patients paid attention to device alerts, bodily sensation remained an important basis for judging postoperative status. Some patients believed that whether they had chest pain, felt uncomfortable, or could perform normal activities was more direct than what was displayed on the device.

P4: “Whether there is something wrong with the body, I know it best myself.

This form of judgment helped patients maintain a sense of certainty in daily life, but it could also lead them to underestimate asymptomatic risk. When there was no obvious bodily discomfort, patients might pay less attention to device-generated abnormality alerts.

P14: “There was no chest pain and no discomfort. Sometimes when it gave an alert, I just found it annoying.”

Some patients repeatedly weighed device alerts against bodily sensations. They neither relied completely on the device nor ignored it entirely, but made judgments by combining their own bodily reactions with physicians' recommendations.

P13: “I look at the watch, but I can't only look at it. I also have to look at how my own body reacts, and then see what the doctor says.”

#### 3.2.3 Device information awaited medical interpretation

##### 3.2.3.1 Seeking physicians' risk translation

Most patients hoped that physicians would explain device alerts. What patients were truly concerned about was not a particular number or abnormality label, but whether the alert was serious, whether it was related to the stent, whether an earlier follow-up visit was needed, and whether medication or lifestyle needed to be adjusted.

P20: “I can see the numbers—what the heart rate is, how much sleep I had. I can read those words. But I don't know whether it is serious or not. When it says abnormal, is it a small problem or something that means I should go to the hospital? ”

Physician confirmation also gave device information a sense of formality. If physicians recognized and explained the alert, patients were more likely to regard it as health information requiring attention. If physicians did not respond, patients might treat it as an ordinary reminder.

P7: “If the doctor says I need to pay attention to this, then I will definitely take it seriously.”

Post-PCI patients particularly hoped that physicians would explain the relationship between alerts, stents, and postoperative status. Without physicians' explanations, patients found it difficult to judge whether device alerts indicated stent-related problems, recurrent ischemia, or the risk of needing medical care again.

P15: “What I most want to ask is not whether this number is a bit high or low. I want to know whether it has anything to do with the stent I had placed.”

##### 3.2.3.2 Needing follow-up response loops

Patients not only needed physicians' explanations, but also hoped to obtain follow-up guidance after device abnormalities. Follow-up timing, medication precautions, exercise intensity, and ways to handle symptoms were the issues for which they most wanted clear responses.

P1: “If the watch shows something abnormal, it would be good if someone could remind me whether I should have a follow-up visit and whether I need to pay attention to my medication. ”

Older patients or those with weaker digital health literacy needed more human follow-up support. For them, device notifications themselves did not necessarily translate into action. Telephone calls, WeChat communication, or outpatient follow-up could better help them understand and manage alerts.

P16: “If a nurse could call and tell me, that would be better than me looking at it by myself.”

Some patients expected obvious abnormalities to trigger proactive contact rather than relying entirely on their own judgment. Only when device alerts were connected with follow-up responses could a closed loop be formed from abnormality detection to guidance.

P6: “If the risk or abnormality is quite obvious, could the hospital contact us proactively? Otherwise, we are not sure ourselves.”

##### 3.2.3.3 Unclear responsibility for device alerts

Some patients were uncertain whether device alerts were recognized by the hospital, and they did not know whether they should consult physicians, nurses, customer service staff, or family members. The lack of a clear responsible party meant that alerts could easily remain within patients' personal worries.

P23: “This alert comes from the watch itself. I don't know whether the hospital recognizes it or not.”

When devices were purchased by patients themselves, their relationship with the hospital diagnosis and treatment system became even more ambiguous. Patients worried that physicians could not see this information and also worried that physicians might consider it to have no clinical value.

P19: “I bought this watch myself. When I mentioned it to the doctor during a previous examination, the doctor told me to rely on the hospital's examinations.”

Unclear responsibility also led to uncertainty about action pathways. After receiving device alerts, patients had more bodily information, but they did not know whether they needed to record it, when to address it, or through which channel they should obtain an explanation.

P11: “It gives alerts, but I don't know who is responsible for this. If I ask the doctor, I may not be able to ask right away. If I ask customer service, they are not doctors.”

##### 3.2.3.4 Choosing to wait and observe amid uncertainty

Even when patients were worried about device alerts, they did not necessarily consult physicians immediately. Some patients believed that going to the hospital because of a single device alert might be overreacting, and they were also concerned about the inconvenience of registration or taking up physicians' time.

P14: “It doesn't feel right to bother the doctor just because the watch beeped once. It feels like making too much of it.”

Some patients first sought help from family members, fellow patients, or online information before deciding whether to seek medical care. Device alerts often first entered the family and informal information networks rather than the formal healthcare system.

P9: “I usually ask my children first and let them see whether it is serious.”

Waiting and observing did not mean that patients ignored device information. Rather, they weighed the frequency of alerts, the severity of symptoms, and the convenience of seeking medical care. Patients were more likely to consult physicians only when alerts occurred repeatedly or were accompanied by obvious discomfort.

P21: “If it only reminds me once, I may watch it first. If it happens for several days in a row, then I need to ask the doctor.”

#### 3.2.4 From self-management to disease burden

##### 3.2.4.1 Strengthening awareness of medication use and follow-up

After seeing device alerts, some patients became aware again that long-term management was still necessary after PCI. Alerts made them realize that symptom relief did not mean they could relax medication use, nor could they postpone follow-up simply because they had no short-term discomfort.

P15: “When I see these alerts, I think that I still cannot stop the medication casually, and I cannot keep delaying follow-up. After all, I had a stent placed, so I still need to pay attention.”

Device alerts also prompted patients to refocus on antiplatelet medication, lipid-lowering medication, and risk factor control. Especially when a longer time had passed after PCI, patients tended to loosen their management, while device alerts could reinforce their awareness of illness again.

P12: “After a long time, people easily relax. When the watch reminds me, I think that I am still a patient, and I still need to take my medicine on time”

Some patients linked insufficient activity, poor sleep, or heart rate changes with their recent management status, reflecting on whether they had become lax in medication use, follow-up, exercise, or rest.

P18: “When I see things like low activity or poor sleep, I also reflect on myself a bit.”

##### 3.2.4.2 Promoting lifestyle restraint

Device alerts prompted some patients to begin placing constraints on daily life, including eating less greasy food, controlling smoking and alcohol use, increasing activity, paying attention to sleep, and maintaining emotional stability. Compared with general health education, individualized alerts more easily made patients feel that the information was directly related to themselves.

P8: “I used to know that I should eat less oily food, but that was just something people said. When I saw that my own data were not good, I felt that this really had something to do with me.”

Some patients adjusted their exercise patterns according to physical activity and post-exercise heart rate. They no longer simply accepted the general recommendation to “exercise appropriately,” but tried to use device displays to judge whether their exercise was sufficient or excessive.

P13: “I pay attention to my heart rate after exercise. If it is too high, I don't dare to increase the amount, I make some adjustments by looking at it.”

Device information also prompted patients to pay attention to sleep and emotions. Some patients linked staying up late, high stress, or poor sleep with cardiac recovery and actively adjusted their daily lives.

P22: “Now I pay attention to not staying up late, and I try to keep my emotions stable. ”

##### 3.2.4.3 Real-life conditions limited action

Although patients knew they needed management, work, family, economic conditions, and dietary environments limited change. Device alerts could enhance management awareness, but they could not automatically solve the practical difficulties of implementation in real life.

P5: “I know I need to control my diet, but I eat outside for work. How could every meal follow the requirements exactly?”

Digital health literacy also influenced the effects of device alerts. Some older patients or patients with lower educational levels relied on family members to check information. When family members were not present, device alerts were difficult to translate into actual management.

P9: “I don't understand what it says. When my children are around, they can help me look at it. If they are not around, I don't even want to wear it myself.”

Long-standing habits also limited change. Dietary preferences, smoking and alcohol use, sedentary behavior, or lack of exercise had persisted for many years. Even though device alerts increased willingness to change, they were difficult to translate immediately into stable behaviors.

P19: “I understand all the reasoning, and the watch also reminds me, but habits of several decades cannot be changed just because I say I will change them.”

##### 3.2.4.4 Continuous monitoring brought fatigue

For patients who had long been in a state of postoperative management, device alerts were sometimes not a source of motivation but a source of pressure. Continuously checking heart rate, sleep, activity, and abnormality alerts reinforced the feeling that they were “always a patient.”

P12: “After the stent was placed, I never really let go of it. I have to take medicine and go for follow-ups, and now the watch shows me all these things every day. Sometimes when I see too much of it, I get annoyed. It feels like I am always managing this disease, as if there is no end.”

After seeing alerts, some patients paid more frequent attention to chest discomfort, heartbeat changes, or activity tolerance. Moderate attention could help identify abnormalities, but excessive observation might amplify mild discomfort and increase anxiety.

P6: “Before I wore it, I didn't feel anything. Now, whenever it reminds me, I feel chest pain.”

If the device only indicated abnormality without explaining the cause, severity, or coping strategies, patients entered a state of “knowing that something is abnormal but not knowing what to do.” In this situation, the device was no longer merely a management tool, but also became a new source of uncertainty.

P20: “It just tells you that something is abnormal, but it doesn't say what to do after that.”

## 4 Discussion

This study showed that when patients with coronary heart disease (CHD) after percutaneous coronary intervention (PCI) encountered information generated by smart bands or smart wrist-worn devices, such as heart rate, heart rhythm, activity, and sleep data, they did not simply accept or reject this information. Instead, they repeatedly judged these alerts in relation to their own symptoms, experiences of stent implantation, memories of previous disease onset, follow-up results, and physicians' explanations. Previous studies have demonstrated that smartwatches and smart bands can record heart rate, provide heart rhythm abnormality alerts, and screen for atrial fibrillation through photoplethysmography (PPG), single-lead electrocardiography (ECG), and other approaches (22), Large-scale studies such as the Apple Heart Study (23) and the Fitbit Heart Study (24) have also suggested that wrist-worn devices have certain potential for identifying irregular heart rhythms and detecting some cases of asymptomatic atrial fibrillation,These studies mainly answer the question of “whether devices can detect certain rhythm abnormalities.” In contrast, the present study further shows that, among post-PCI patients, the more critical patient-side questions are how patients understand these alerts after seeing them, whether they trust them, whether they take action, and whether these alerts can be interpreted and integrated by the healthcare system (25).

Participants in this study used smart wrist-worn devices with different monitoring functions and levels of technological sophistication. Devices with electrocardiographic functions may provide more detailed rhythm-related information, whereas devices limited to heart-rate monitoring generally provide simpler measurements and alerts. Such differences may influence patients' trust in device-generated information, their levels of reassurance or anxiety, and their subsequent responses. Therefore, the findings should be interpreted in relation to the specific functions and alert characteristics of the devices used.

First, this study found that smart wrist-worn devices transformed patients' access to heart rate, heart rhythm, and health-related data from “intermittent contact” to “continuous exposure.” Existing studies on cardiac rehabilitation and remote monitoring have mostly interpreted the role of wearable devices from the perspective of behavioral management, such as recording physical activity and heart rate changes, increasing awareness of exercise and lifestyle management, and thereby supporting secondary prevention (26) The present findings are consistent with this perspective, but further reveal that continuous exposure to device data does not lead only to a one-way process of “strengthened management.” Patients repeatedly encountered heart rate, sleep, or abnormality alerts in non-medical settings. Such information was often separated from physicians' explanations and clinical contexts, requiring patients to assign meaning to it by themselves (27). Some patients were able to use this information to maintain attention to medication use, follow-up, and lifestyle management, whereas others repeatedly reinforced their perception of being in a disease state because they lacked an interpretive framework.

Second, this study suggests that device-generated information was reinterpreted by post-PCI patients within their previous disease experiences. Existing research on wearable devices has mainly focused on atrial fibrillation, rhythm abnormalities, or heart rate recording, with relatively limited attention to how patients with CHD after PCI understand such alerts (26). This study found that when patients saw elevated heart rate alerts or rhythm reminders, they often associated them with previous chest pain, emergency admission, stent implantation, and concerns about “whether the blood vessel was blocked again” (28). Although smart bands cannot determine whether a stent is patent or diagnose coronary restenosis, patients do not necessarily interpret alerts according to the technical boundaries of device functions. For patients who have undergone PCI, the stent represents a concrete bodily memory. The same heart rate or rhythm alert may be only a health reminder for ordinary users, but for post-PCI patients it may be assigned meanings related to recurrence or stent safety.

Third, this study found that bodily sensations remained an important basis for patients to judge device alerts. Previous studies have indicated that wrist-worn devices can capture some asymptomatic or intermittent rhythm abnormalities, which is one advantage of continuous monitoring compared with a single examination (29). However, from the perspective of patient experience, an “asymptomatic abnormality” is not necessarily understood directly as risk. If patients had no chest pain, palpitations, shortness of breath, or reduced activity tolerance, they often suspected that the alert might be caused by improper wearing, measurement error, or ordinary physiological fluctuation. Conversely, when device alerts occurred together with chest tightness, palpitations, or post-exercise discomfort, patients were more likely to believe that the device “made sense” (30). Existing research emphasizes that continuous monitoring can detect abnormalities missed by symptoms. The present study further suggests that even when devices can detect abnormalities, whether patients take them seriously is still shaped by bodily sensations and previous experiences.

Although bodily sensations provided patients with an immediate and familiar basis for interpreting device alerts, relying on the absence of discomfort to dismiss an alert may carry clinical risks. Myocardial ischemia may occur without typical symptoms, and the absence of chest pain, palpitations, or other perceived discomfort should not be interpreted as evidence that cardiovascular risk is absent. This pattern may partly reflect patients' limited understanding of the relationship between symptoms and underlying coronary pathology. At the same time, consumer-grade wearable devices cannot independently diagnose myocardial ischemia, coronary restenosis, or stent-related complications. Therefore, neither bodily sensations nor device-generated alerts should be used alone to determine cardiovascular risk, and patients require clear guidance regarding when professional clinical assessment is needed.

This study also resonates with research on patient-generated health data. Previous studies have noted that health data generated by wearable devices can provide healthcare professionals with information that is closer to patients' everyday lives, but their clinical use still faces challenges related to data quality, responsibility for interpretation, time burden, and workflow integration (31). From the patient perspective, this study further shows that these challenges also exist on the patient side. Patients can see numbers and alerts, but they may not know whether these are serious; they can save data, but may not know whether physicians recognize it; they may want to consult physicians, but worry about overreacting or not know whom to ask. Therefore, information from wrist-worn devices does not automatically translate into medical action (32). Only when device alerts are incorporated into outpatient communication, nurse-led follow-up, or digital follow-up processes can wearable-generated information be transformed from data requiring independent patient interpretation into actionable management recommendations (16).

Physicians' explanations played an evident role in this study. Existing digital health research often emphasizes patient empowerment and self-management. However, the present study shows that, at least among post-PCI patients, empowerment does not mean transferring risk judgment entirely to patients. What patients hoped physicians would explain was not the technical meaning of every number, but whether these alerts were related to their CHD, post-PCI status, and experience of stent implantation (33). In this context, smart bands are more suitable as auxiliary materials for clinician–patient communication than as independent diagnostic tools or substitutes for follow-up examinations.

In addition, this study showed that smart wrist-worn devices had a dual effect on self-management. Existing cardiac rehabilitation studies usually emphasize the role of wearable devices in promoting exercise, adherence, and remote management. This study also found that patients reported that device alerts could make them pay more attention to medication use and follow-up, dietary control, increased activity, sleep, and emotional regulation (34). However, continuous exposure to device data may also increase disease burden. In addition to long-term medication use and follow-up, some patients also had to continuously face heart rate, sleep, activity, and abnormality alerts, which could contribute to a persistent sense of illness burden. In particular, when devices only indicated abnormalities without explaining the cause, severity, or coping strategies, patients might feel increasingly unsettled the more they looked at the data. Therefore, digital information is not inherently equivalent to empowerment. In the absence of explanation and response, it may also transfer more uncertainty and responsibility for judgment to patients.

Based on these findings, attention should be paid to the clinical boundaries and patient-side interpretive needs when smart bands or smart wrist-worn devices are used in post-PCI management. On the device side, alerts should not simply use terms such as “abnormal,” “increased risk,” or color changes to notify patients. Instead, they should, as far as possible, distinguish between everyday fluctuations, changes that require observation, and situations in which contacting healthcare professionals is recommended (35). In follow-up and health education, healthcare professionals may also proactively ask patients whether they use smart bands, how they understand heart rate and rhythm alerts, and whether these alerts have caused concern. For older patients, those with lower digital literacy, or those who rely on family members to view data, additional support is needed, such as family involvement, telephone follow-up, or nurse-led explanation. It is particularly important to explain to patients that smart bands can assist in observing daily status, but cannot directly determine stent patency, coronary restenosis, or recurrence of cardiovascular events (33).

Healthcare services should consider establishing a structured response pathway for patients seeking consultation because of wearable-generated information. Initial assessment may include clarifying the type and function of the device, reviewing the timing and pattern of alerts, identifying accompanying symptoms, and considering the patient's clinical history and overall cardiovascular risk. When clinically indicated, wearable-generated information should be verified through standard clinical assessment rather than treated as diagnostic evidence in isolation.

Wearable devices may be selectively incorporated into routine post-PCI care as supplementary sources of patient-generated health information rather than as replacements for clinical evaluation. Such integration requires clear triage criteria, defined responsibilities among physicians, nurses, and follow-up teams, appropriate documentation of relevant device information, and a closed-loop process for communicating assessment results and follow-up recommendations to patients. This approach may help reduce unnecessary anxiety caused by isolated alerts while decreasing the likelihood that potentially important information is overlooked.

## 5 Conclusions

This study found that when patients with coronary heart disease after percutaneous coronary intervention (PCI) used smart wrist-worn devices, they did not simply accept the heart rate, heart rhythm, and health-related data generated by the devices. Instead, they compared this information with their own bodily sensations, previous disease experiences, experiences of stent implantation, follow-up results, and explanations from healthcare professionals in order to judge its risk-related meaning. Smart wrist-worn devices may prompt patients to pay greater attention to medication use, follow-up, and lifestyle management. However, in the absence of medical interpretation and follow-up integration, they may also increase uncertainty and disease burden. Therefore, in post-PCI management, smart wrist-worn devices should be positioned as auxiliary tools for daily status observation and clinician–patient communication, rather than as substitutes for clinical examinations or professional judgment. Future efforts should further clarify the interpretive boundaries of device-generated information and establish clearer pathways for stratified guidance and follow-up response.

## 6 limitations

This study has several limitations. First, all participants were recruited from a single tertiary hospital in China. The cultural and healthcare-system context may have influenced participants' reliance on healthcare professionals for interpreting device-generated information, their expectations regarding professional authority, and their perceptions of responsibility for responding to wearable-related alerts. These factors may have particularly shaped the findings concerning the need for medical interpretation and unclear responsibility for device alerts. Therefore, the transferability of these findings to other countries and healthcare systems should be considered with caution, especially where attitudes toward technology, patient autonomy, healthcare authority, and responsibility for consumer-generated health data differ.

Second, although both previous and current users of smart wrist-worn devices were eligible, the study mainly included patients who were willing and able to describe their experiences with wearable-generated health information. Patients who discontinued use shortly after adoption, rejected the technology, or stopped using it because of anxiety, usability difficulties, or limited perceived value may have been underrepresented. Consequently, the findings may not represent all post-PCI patients who have attempted to use smart wrist-worn devices. Future studies should include more former users and patients who discontinued use to better understand barriers, burdens, and reasons for discontinuation.

Third, differences in device brands, models, monitoring functions, and alert formats may have influenced participants' trust, emotional responses, and interpretations of device-generated information. However, this study was not designed to compare experiences across specific device categories.

Finally, this study focused on patients' interpretations and responses and did not assess device accuracy or the effects of device use on clinical outcomes, medication adherence, follow-up attendance, or lifestyle behaviors. Future research could combine device-use records, clinical data, and interviews with patients and healthcare professionals to further examine how wearable-generated information may be incorporated into post-PCI care.

## Data Availability

The original contributions presented in the study are included in the article/Supplementary Material, further inquiries can be directed to the corresponding author.
