# Supplementary material for: Between algorithmic alerts and bodily sensations: a qualitative study of post-PCI patients' experiences with wrist-worn heart health information
Source: Front Cardiovasc Med. 2026 Oct 19;13:1926958. doi: 10.3389/fcvm.2026.1926958 (PMC13724597; doi:10.3389/fcvm.2026.1926958)
Supplement: Supplementary file 1 [file Table1.docx]

| Table 1. Participant Characteristics | | | | | | | | | | | |
| --- | --- | --- | --- | --- | --- | --- | --- | --- | --- | --- | --- |
| ID | Sex | Age | Educational Level | Time Since PCI | PCI-Related Clinical Background | Major Comorbidities | Use of β-Blockers | Type of Smart Wrist-Worn Device | Duration of Device Use | Main Indicators of Concern | Previous Abnormality Alert |
| P1 | Male | 62 | Junior high school | 8 months after PCI | PCI after acute coronary syndrome | Hypertension | Yes | Huawei smartwatch | 7 months | Heart rate, sleep, step count | Yes |
| P2 | Female | 58 | High school/technical secondary school | 1 year and 2 months after PCI | PCI after acute myocardial infarction | Hypertension, diabetes mellitus | Yes | Xiaomi smart band | 1 year | Heart rate, step count, sleep | Yes |
| P3 | Male | 66 | Junior high school | 2 years after PCI | Elective PCI for stable CHD | Hypertension | Yes | Huawei smartwatch | 2 years | Heart rate, blood oxygen, physical activity | Yes |
| P4 | Male | 54 | Junior college | 6 months after PCI | PCI after unstable angina | Hyperlipidemia | Yes | Apple Watch | 6 months | Heart rate, electrocardiography, physical activity | No |
| P5 | Female | 61 | Junior high school | 10 months after PCI | PCI after acute coronary syndrome | Hypertension | Yes | Huawei smart band | 9 months | Heart rate, sleep | Yes |
| P6 | Male | 70 | Primary school | 3 years after PCI | PCI after acute myocardial infarction | Hypertension, diabetes mellitus | Yes | Xiaomi smart band | 2 years | Heart rate, step count | Yes |
| P7 | Male | 59 | Bachelor’s degree | 1 year after PCI | PCI after unstable angina | Hyperlipidemia | Yes | Apple Watch | 1 year | Heart rate, electrocardiography, sleep | Yes |
| P8 | Female | 64 | High school/technical secondary school | 1 year and 6 months after PCI | Elective PCI for stable CHD | Hypertension | Yes | Huawei smartwatch | 1 year | Heart rate, blood oxygen, sleep | No |
| P9 | Male | 57 | Primary school | 5 months after PCI | PCI after acute coronary syndrome | Hyperlipidemia | Yes | Huawei smartwatch | 5 months | Heart rate, physical activity | Yes |
| P10 | Male | 72 | Primary school | 4 years after PCI | PCI after acute myocardial infarction | Hypertension, diabetes mellitus | Yes | Xiaomi smart band | 3 years | Heart rate, step count, sleep | Yes |
| P11 | Female | 55 | Bachelor’s degree | 9 months after PCI | Elective PCI for stable CHD | Hyperlipidemia | No | Apple Watch | 8 months | Heart rate, electrocardiography, sleep | Yes |
| P12 | Male | 68 | Junior high school | 2 years and 6 months after PCI | PCI after acute coronary syndrome | Hypertension | Yes | Huawei smart band | 2 years | Heart rate, blood oxygen | Yes |
| P13 | Male | 60 | High school/technical secondary school | 1 year and 3 months after PCI | PCI after unstable angina | Hypertension, hyperlipidemia | Yes | Huawei smartwatch | 1 year | Heart rate, sleep, physical activity | No |
| P14 | Female | 63 | Junior high school | 7 months after PCI | Elective PCI for stable CHD | Diabetes mellitus | No | Xiaomi smart band | 6 months | Heart rate, step count | Yes |
| P15 | Male | 52 | Bachelor’s degree | 4 months after PCI | PCI after acute coronary syndrome | Hyperlipidemia | Yes | Apple Watch | 4 months | Heart rate, electrocardiography, physical activity | Yes |
| P16 | Male | 69 | Junior high school | 3 years and 6 months after PCI | PCI after acute myocardial infarction | Hypertension | Yes | Huawei smartwatch | 3 years | Heart rate, blood oxygen, sleep | Yes |
| P17 | Female | 56 | Junior college | 1 year after PCI | PCI after unstable angina | Hypertension | Yes | Huawei smart band | 1 year | Heart rate, sleep | Yes |
| P18 | Male | 65 | High school/technical secondary school | 2 years after PCI | Elective PCI for stable CHD | Hypertension, diabetes mellitus | Yes | Xiaomi smart band | 1 year and 8 months | Heart rate, step count, sleep | No |
| P19 | Male | 74 | Primary school | 5 years after PCI | PCI after acute myocardial infarction | Hypertension, diabetes mellitus | Yes | Huawei smartwatch | 2 years | Heart rate, blood oxygen | Yes |
| P20 | Female | 59 | Junior college | 11 months after PCI | PCI after unstable angina | Hyperlipidemia | Yes | Apple Watch | 10 months | Heart rate, sleep, electrocardiography | Yes |
| P21 | Male | 67 | Junior high school | 2 years and 3 months after PCI | Elective PCI for stable CHD | Hypertension | Yes | Huawei smart band | 2 years | Heart rate, step count | No |
| P22 | Male | 53 | Bachelor’s degree | 6 months after PCI | PCI after acute coronary syndrome | Hyperlipidemia | Yes | Huawei smartwatch | 6 months | Heart rate, physical activity, sleep | Yes |
| P23 | Female | 71 | Primary school | 4 years and 6 months after PCI | PCI after acute myocardial infarction | Hypertension, diabetes mellitus | Yes | Xiaomi smart band | 3 years | Heart rate, step count, sleep | Yes |
