# Supplementary material for: Between algorithmic alerts and bodily sensations: a qualitative study of post-PCI patients' experiences with wrist-worn heart health information
Source: Front Cardiovasc Med. 2026 Oct 19;13:1926958. doi: 10.3389/fcvm.2026.1926958 (PMC13724597; doi:10.3389/fcvm.2026.1926958)
Supplement: Supplementary file 2 [file Table2.docx]

**Use and Structure of the Interview Guide**

The interview guide was used flexibly within a semi-structured interview format rather than as a structured questionnaire with fixed response categories. It followed a progressive structure, moving from broad, open-ended exploration to more focused inquiry. Questions 1–4 were designed to elicit participants’ accounts of their device-use experiences, the information they routinely attended to, their interpretations and responses to abnormal alerts, and the relationship between device-generated information and bodily sensations. Questions 5–9 subsequently explored more specific dimensions based on these initial narratives, including previous illness and treatment experiences, perceptions of post-PCI risk, effects on self-management, and expectations for healthcare support.

Limited examples were included in selected questions to help participants with different ages, educational backgrounds, and levels of familiarity with medical and digital health terminology understand the intended scope of the questions. These examples were not treated as predetermined response categories. Participants were encouraged to confirm, reject, modify, or expand beyond the examples according to their own experiences, and follow-up questions were adapted flexibly to their responses.

| **Interview Guide** | |
| --- | --- |
| 1 | Under what circumstances did you start using a smartwatch or smart band? Was it purchased by yourself, recommended by family members, or recommended by healthcare professionals? |
| 2 | What information from the device do you usually pay most attention to, such as heart rate, heart rhythm alerts, sleep, step count, physical activity, blood oxygen, or electrocardiography records? |
| 3 | When the device indicates abnormalities in heart rate, heart rhythm, sleep, physical activity, or blood oxygen, how do you usually understand and respond to these alerts? |
| 4 | When the device data are consistent or inconsistent with your own bodily sensations, which do you trust more? Why? |
| 5 | Do you associate device alerts with your PCI experience, stent implantation, possible re-occlusion of blood vessels, or follow-up results? How is this association formed? |
| 6 | Has the smart wrist-worn device changed your understanding of your physical condition and disease risk after PCI? |
| 7 | What help, difficulties, or burdens have these device-generated information brought to you in relation to medication use, follow-up, exercise, sleep, and lifestyle management? |
| 8 | When the device generates an abnormality alert, how would you expect physicians, nurses, or the follow-up team to explain and respond to it? |
| 9 | In your view, which device alerts require self-observation, which situations require consultation with healthcare professionals, and which situations require timely medical care? |

| **Development Process of the Interview Guide** | | | |
| --- | --- | --- | --- |
| **Stage** | **Procedure** | **Main consideration** | **Outcome** |
| Identification of interview domains | Study objectives and relevant literature were reviewed | Coverage of post-PCI experiences, wearable information interpretation, and self-management | Nine preliminary content domains were identified |
| Drafting of questions | Questions were formulated according to the nine domains | Open-ended wording, everyday language, and progression from general to specific experiences | An initial interview guide was developed |
| Expert review | Experts reviewed the draft guide | Relevance, clarity, neutrality, sequencing, and comprehensibility | Questions were revised, merged, or reordered |
| Finalization | The research team discussed the revised guide | Avoidance of duplication and excessive prompting | Nine open-ended questions with optional probes were finalized |

| **Domains and Rationale of the Interview Questions** | | |
| --- | --- | --- |
| **Question** | **Content domain** | **Purpose** |
| Q1 | Device adoption | To explore why and under what circumstances participants began using the device |
| Q2 | Information attended to | To identify the types of wearable-generated information participants routinely monitored |
| Q3 | Interpretation and response | To explore how participants understood and acted on abnormal alerts |
| Q4 | Alerts and bodily sensations | To examine how participants handled agreement or disagreement between device information and bodily experiences |
| Q5 | Previous illness and treatment experiences | To explore how prior disease, PCI, treatment, and follow-up experiences shaped alert interpretation |
| Q6 | Postoperative risk perception | To examine whether wearable use changed participants’ understanding of recovery and cardiovascular risk |
| Q7 | Self-management effects | To explore perceived effects on medication use, follow-up, exercise, sleep, and lifestyle management |
| Q8 | Benefits and burdens | To identify support, anxiety, fatigue, or management burden associated with wearable use |
| Q9 | Healthcare support needs | To explore expectations for physicians, nurses, and follow-up services |
